# The American Medical Association

**Published:** 1872-11-15

**Authors:** 


### MEDICAL EXAMINER.

*A Semi-Monthly Journal of Medical Sciences.*

EDITED BY

N. S. DAVIS, M. D., AND F. H. DAVIS, M. D.

Chicago, November 15th, 1872.

### EDITORIAL.

### THE AMERICAN MEDICAL ASSOCIATION.

In a previous number of the EXAMINER we alluded to the gratifying fact that several of those medical journals which had found most fault with the practical workings of the American Medical Association, had, at last, ventured to make suggestions for its improvement; and we promised to give the same a candid examination. Those suggested by the New York *Medical Record* were copied in full in our last issue. They were briefly: *First*, to exclude reporters for the daily secular press from the annual meetings; *Second*, to have the same delegates elected by the State and local societies from year to year; *Third*, to abandon the publication of an annual volume of transactions; and, *Fourth*, to have all the important work of the Association done by "large committees fairly chosen."

The first of these suggestions is worthy of a careful consideration. The practice which has been carried out more fully for the last ten years, of allowing the indiscriminate attendance of ordinary newspaper reporters, with their common habit of giving an exaggerated or sensational coloring to whatever would be most likely to attract the attention of their non-professional readers, coupled with their entire neglect of the more scientific and important part of the work of each meeting, has certainly served each year to place unduly prominent before the public every question of an ethical or merely miscellaneous character, and thereby create a false impression on the public mind. The meeting at San Francisco affords a good illustration of this fact. During the four days that the Association was in session in that city, the discussions concerning the constitutional amendment, and collateral questions involving the rights of women in the profession, occupied an aggregate of about *three* hours. Yet the almost universal impression created throughout the country by the newspaper reports, was that most of the time occupied by the Association in that city was spent in "wrangling about women." Such always will be the result, if ordinary reporters, themselves ignorant of everything pertaining to medicine, are allowed to attend and report freely the miscellaneous doings of the morning sessions, and entirely neglect the more important work of the sections in the afternoon. But the mere exclusion of newspaper reporters, without providing some substitute, would be productive of inconveniences that would be felt by almost every member. It is very desirable that an abstract of the doings of each day should be before the members on the following morning. It not only affords opportunity for correcting errors, but it facilitates in many ways the transaction of business. We have thought that, if the committee of arrangements were instructed to employ, at the expense of the Association, one thoroughly skilful professional reporter for the morning sessions, and one for each of the organized sections in the afternoon, and have them act in such concert, under one leader, that the result of their united labor would appear in a printed slip early each morning—printing a sufficient number of copies to supply not only the members of the Association, but also the editors of medical periodicals, and such local newspapers as choose to use them—it might greatly advance the interests of the Association. Such a plan, by including a fair abstract of all the facts and discussions elicited by the papers and reports presented in the several sections, would not only avoid the false and one-sided coloring given to the doings of each meeting by the present method, but it would also furnish a most valuable addition to the matter in our transactions. To render the plan efficient, the committte of arrangements should also provide a committee of one or two competent physicians to read and revise the proofs as furnished by the reporters. It will be remembered, that at the last annual meeting a proposition was made and referred to a special committee, to give the permanent secretary a salary of $1,000 per annum. We suggest that considerably less than half this sum, annually, would enable the committee of arrangements for each meeting to employ the necessary corps of reporters and committee for revision of proofs, and furnish for each member, every morning, a simple slip or sheet containing a complete abstract of the doings of the previous day, in all the departments of the Association. If so, it would lighten the labor of the Secretary, whose traveling, postage and stationary expenses are already provided for, and would be a much more profitable investment of the funds than in the creation of a salaried officer. Will our friend of the *Medical Record* aid us in perfecting and securing the adoption of such a plan at the next meeting?

The second proposition, that State and local societies should elect the same delegates to represent them several successive years, that they may become more familiar with the rules and modes of transacting business in the Association, is a good one; but it is a matter so completely within the control of the local societies, that we need make no further comments on it in this connection.

The third proposition, which is to abandon totally the publication of an annual volume of transactions, is, to say the least, of doubtful propriety. It is true that most of the valuable reports and papers read to the Association might be published in the various medical periodicals, and in separate monographs, and thereby, perhaps, reach a larger number of readers than if issued in a volume together. It is also true that some worthless matter finds its way into almost every volume of transactions. But we cannot agree with our cotemporary, that these volumes are so utterly worthless as he represents. We have a complete set of them, and must acknowledge that there is not one which does not contain matter of very great practical value, and that, too, sufficient to repay the careful reader ten times its cost. We fear those editors who speak so disparagingly of the transactions have, themselves, taken the trouble to read but very few of their pages. We would not favor the abolition of the transactions. We are quite sure they might be reduced in size, and much increased in value, if the system of reporting we have suggested were adopted, and the several sections would strictly follow, in the disposal of the reports and papers presented, the present rules and ordinances adopted for their guidance. And if each medical journal in the country would publish these rules two or three months before each annual meeting, and urge upon their readers the necessity of strictly complying with them in their action upon reports and papers, they would do more good than by all the editorial grumbling that has been indulged in during the last ten years.

The fourth proposition of the *Record*, is to have all the important work of the Association done by *large committees, fairly chosen.* The editor does not tell us *how large* he would have each committee; how many he would have; or how he would secure their being "fairly chosen." The proposition is hardly definite enough to enable us to judge of its practicability. We doubt whether it is possible to devise a series of large committees that would be as efficient in practical work, as the present sections, if the organization and duties of the latter were better understood, and the rules adopted for their government better observed. If the committee of arrangements for the next meeting in St. Louis, will take the trouble to read the constitution and by-laws, so far as relates to the sections, and provide each one with a suitable room that can be found, to meet in, and the several medical periodicals will early call the attention of their readers to the necessity of complying with the regulations adopted for governing the sections, by publishing them, there will be little occasion for criticising the results of their work.

---

THE Michigan Medical Society admits female practitioners to its membership.

